# Sleep Apnea and Cardiovascular Disease: Risks, Mechanisms, Complications, and Management

**DOI:** 10.31083/RCM50734

**Published:** 2026-06-22

**Authors:** Noyan Ramazani, Sahar Mahani, Mohamad Mubder, Darren Nguyen, Nazanin Houshmand, Sigurd Hartnett, Tillmann Cyrus, Tahir Tak

**Affiliations:** ^1^Department of Internal Medicine, University of Nevada Las Vegas, Kirk Kerkorian School of Medicine, Las Vegas, NV 89102, USA; ^2^Department of Cardiovascular Medicine, University of Nevada Las Vegas, Kirk Kerkorian School of Medicine, Las Vegas, NV 89102, USA; ^3^Department of Cardiovascular Medicine, Corpus Christi Medical Center, Corpus Christi, TX 78411, USA; ^4^Department of Cardiology, VA Southern Nevada Healthcare System, North Las Vegas, NV 89086, USA

**Keywords:** sleep apnea, atrial fibrillation, heart failure, pulmonary hypertension, sudden cardiac arrest, cardiovascular disease, continuous positive airway pressure therapy, intermittent hypoxia

## Abstract

Sleep apnea is a common sleep disorder characterized by recurrent episodes of breathing cessation and resumption during sleep. The three main types are obstructive sleep apnea (OSA), central sleep apnea (CSA), and mixed sleep apnea (MSA), the latter of which is often used interchangeably with complex sleep apnea (CompSA). OSA, the most prevalent form, results from upper airway obstruction during sleep. Globally, nearly one billion adults aged 30–69 are affected by OSA, with approximately 425 million having moderate to severe disease, making sleep apnea a major global health burden affecting roughly one in eight individuals. Sleep apnea is associated with numerous serious comorbidities and health complications, most notably cardiovascular disease (CVD). The adverse consequences of sleep apnea on cardiovascular health arise from intermittent hypoxia and inflammation, which contribute to hypertension, coronary artery disease (CAD), heart failure (HF), cardiac arrhythmias such as atrial fibrillation (AF), pulmonary arterial hypertension (PAH), stroke or cerebrovascular accident (CVA), and sudden cardiac arrest (SCA), potentially culminating in sudden cardiac death (SCD). Effective treatment of sleep apnea and its deleterious cardiovascular sequelae is critical to preventing ongoing harm. Thus, this review discusses institutionally sponsored and guideline-directed medical therapies aimed at reducing mortality in patients with sleep apnea and CVD, including continuous positive airway pressure (CPAP), pharmacological strategies, and other interventions to manage sleep apnea and its cardiovascular-related comorbidities. This review also highlights screening for sleep apnea using the STOP-BANG questionnaire and emphasizes the importance of a multidisciplinary management approach, which is crucial for preventing further physiological damage that can lead to CVD.

## 1. Introduction

**Fig. 1.**
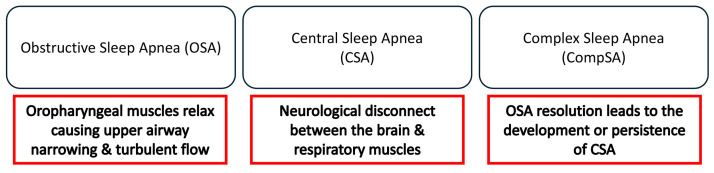
**The three main types of sleep apnea and the associated underlying physiological causes**.

According to the National Heart, Lung, and Blood Institute (NHLBI), a center within the National Institutes of Health (NIH), sleep apnea is a condition in which the breathing of a person repeatedly stops and restarts during sleep. The American Academy of Sleep Medicine recognizes three main types of sleep apnea (Fig. 1): obstructive sleep apnea (OSA), central sleep apnea (CSA), and complex sleep apnea (CompSA), also termed mixed sleep apnea (MSA). The subtype complex sleep apnea syndrome (CompSAS) is characterized by the emergence or worsening of CSA during treatment with continuous positive airway pressure (CPAP) for OSA, whereas MSA is diagnosed when both OSA and CSA are present simultaneously [1]. OSA is the most commonly diagnosed among the three main types.

For OSA specifically, various studies have investigated soft tissue morphology, craniofacial structure, and orthodontic variants, features commonly observed in the general population, as key etiological factors [2]. Sleep apnea is diagnosed when there are more than five apneic episodes per hour of sleep or recurrent pauses in respiratory airflow lasting longer than 10 seconds [3]. The sleep apnea–hypopnea index (AHI), defined as the number of apneic and hypopneic episodes per hour of sleep, is used to quantify disease severity. Apnea is categorized into four groups (Table 1): normal (AHI <5), mild sleep apnea syndrome (5 < AHI < 15), moderate sleep apnea syndrome (15 < AHI < 30), and severe sleep apnea syndrome (AHI >30) [3,4].

**Table 1.** **The four categories of sleep apnea are defined by the apnea–hypopnea index (AHI). The AHI is calculated as the total number of apneas and hypopneas during the night divided by the total number of hours the subject slept**.

Sleep apnea severity	Apnea–hypopnea index (AHI)	Description
Normal (no sleep apnea)	<5	Breathing events are within the normal range
Mild sleep apnea	5–15	No symptoms or only mild daytime sleepiness
Moderate sleep apnea	15–30	Occasional daytime sleepiness during activities that require attention and focus
Severe sleep apnea	>30	Frequent daytime sleepiness that interferes with normal daily activities

Polysomnography is the gold standard for diagnosing sleep apnea. Polysomnography continuously and simultaneously measures multiple electrophysiological and respiratory parameters with high precision [3]. Sleep apnea can occur at any phase of the sleep cycle; however, this disorder is more commonly detected during rapid eye movement (REM) and non-rapid eye movement (NREM) sleep, with most events occurring in the N2 and N3 stages. This repeated stimulation and suppression of the respiratory drive, in conjunction with the sleep cycle, often leads to fatigue due to inadequate oxygenation and systemic perfusion, with blood failing to adequately reach vital tissues and organ systems. Persistent oxygen starvation leads to excessive daytime sleepiness and, if left untreated, can result in serious complications, including cardiovascular disease (CVD).

## 2. Methods

A comprehensive literature search was conducted in PubMed, Embase, and Google Scholar. The following keywords were used: “sleep apnea”, “cardiovascular disease”, “complications”, “pathogenesis”, “CPAP”, and “medical management”. Only English-language publications were included, and most of the studies consulted and used to guide this review were published between 2000 and 2025.

## 3. Epidemiology and Risk Factors

OSA is one of the most common sleep disorders worldwide, affecting an estimated 936 million adults aged 30–69 years. In the United States of America (USA), prevalence varies depending on the diagnostic criteria used. Indeed, using the AHI criterion of >5 events/hour, the prevalence is estimated at 33.9% in men and 17.4% in women; using a criterion of >15 events/hour, the prevalence is 13% and 6%, respectively [5]. Other studies have reported that the prevalence of sleep apnea, specifically OSA, in middle-aged adults is approximately 34% in men and 17% in women [6]. OSA is substantially underdiagnosed in the USA. It is estimated that 82% of men and 93% of women with OSA are unaware of their condition. Sleep-disordered breathing (SDB) and CVD share multiple risk factors, including male sex, older age, and obesity.

SDB is 2–4-fold more prevalent in men than in women. This sex difference is attributed to anatomical variations: men tend to have a longer, softer oropharynx; a larger, more posteriorly positioned tongue; greater upper airway fat deposition and narrowing than women, all of which increase susceptibility to large-airway collapse. Upper airway collapsibility, determined by the pharyngeal critical closing pressure, is lower in women [7]. OSA is more prevalent in older individuals, likely due to increased stiffness of the oropharyngeal structures and a higher burden of comorbidities. Anatomic abnormalities such as enlarged tonsils, increased neck circumference, and a large tongue are recognized risk factors for OSA. Sex is another important non-modifiable risk factor. Women generally have lower ventilatory drive and cortical arousal, shorter respiratory events, a higher proportion of hypopneas relative to apneas, and less oxygen desaturation during respiratory events [8]. Studies have shown that men exhibit a greater ventilatory response to apneas during NREM sleep but develop more pronounced hypoventilation upon returning to sleep, which may contribute to more severe symptoms of sleep apnea in men [5]. In women, sleep apnea is more prevalent after menopause, unless hormone replacement therapy is used [7].

Maladaptive lifestyle habits such as alcohol use and smoking can increase the risk of OSA. Alcohol was found to relax the oropharyngeal muscles, promoting upper airway obstruction, while smoking can increase upper airway inflammation, affecting the quality of breathing. Meanwhile, obesity is one of the most important risk factors for OSA and can both predispose to and be exacerbated by OSA, creating a vicious cycle [9]. Leptin, a hormone that plays a crucial role in regulating body weight and appetite, correlates with the severity of OSA. Studies have shown elevated leptin levels in both patients with OSA and those with obesity [10]. The prevalence of OSA can reach up to 77% among obese patients requiring bariatric surgery, and polysomnographic testing is recommended for all these patients. Finally, OSA is highly prevalent in patients with end-stage renal disease, acute cerebrovascular accident (CVA), and heart failure (HF) through various pathophysiological mechanisms. Thus, appropriate identification and treatment of OSA in these populations improves both survival and quality of life [11,12].

## 4. Mechanistic Link Between Sleep Apnea and Cardiovascular Disease

**Fig. 2.**
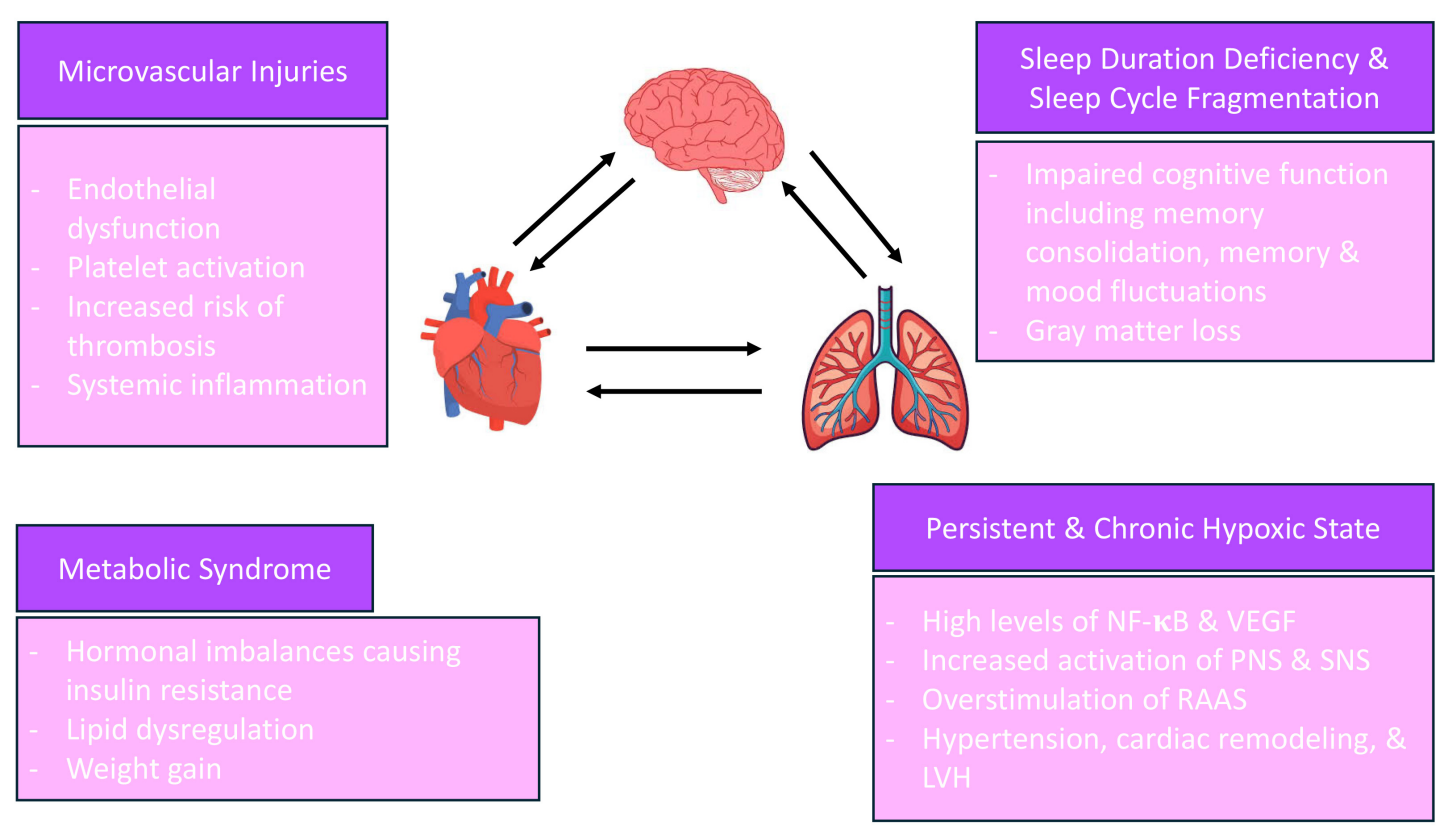
**The pathophysiological interplay between the brain, heart, and lungs in the context of sleep apnea and associated disease processes**. NF-κB, nuclear factor kappa-light-chain-enhancer of activated B cells; VEGF, vascular endothelial growth factor; PNS, parasympathetic nervous system; SNS, sympathetic nervous system; RAAS, renin–angiotensin–aldosterone system; LVH, left ventricular hypertrophy.

There is a strong pathophysiological relationship between SDB, such as sleep apnea, and CVD, which affects the brain, heart, and lungs (Fig. 2). A well-established association exists between SDB and systemic hypertension, and additional adverse cardiovascular outcomes have been identified, including an increased risk of stroke or CVA, coronary artery disease (CAD), and HF [13]. OSA is also strongly associated with metabolic disorders, which are also major risk factors for CVD [14]. Moreover, OSA is a potent risk factor for heart failure with reduced ejection fraction (HFrEF), but the association between OSA and heart failure with preserved ejection fraction (HFpEF) is less well recognized; both conditions are common worldwide [15].

**Fig. 3.**
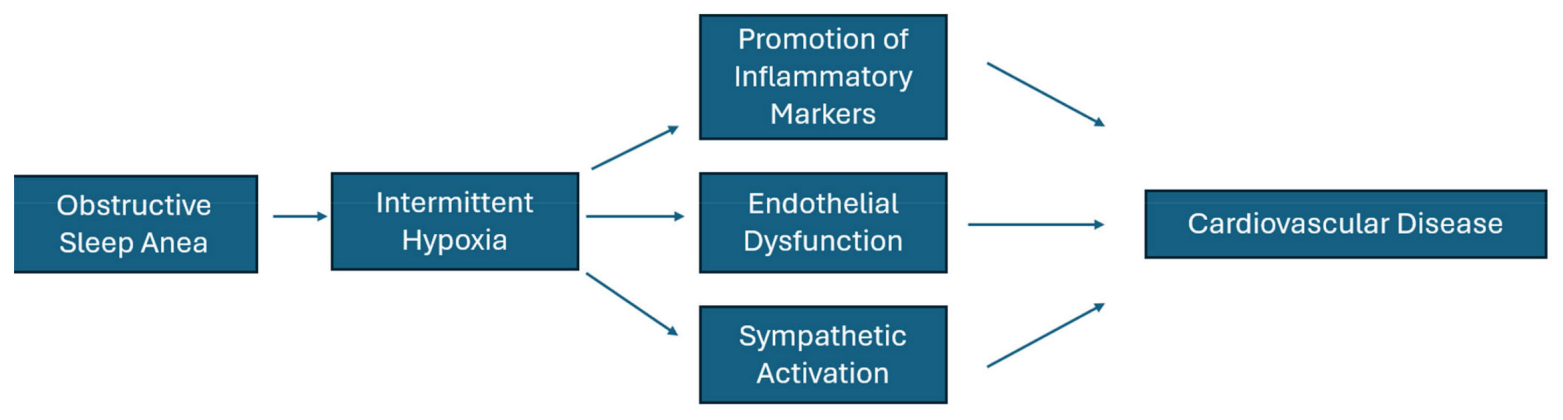
**Summary of the pathophysiology of cardiovascular disease (CVD) in OSA**. OSA, obstructive sleep apnea.

In both HFrEF and HFpEF, intermittent hypoxia, sympathetic overstimulation, renin–angiotensin–aldosterone system (RAAS) dysregulation, and a systemic inflammatory state, combined with oxidative stress, all contribute to the development of multiple CVD comorbidities (Fig. 3) [15]. The result of uncontrolled OSA on the cardiovascular system is myocardial fibrosis, structural changes in collagen and titin, and increased myocardial stiffening, all of which contribute to overall pathologic cardiac remodeling [15]. This cardiac remodeling has both macro- and microscopic consequences, including cardiac chamber dilation that promotes HF and fibrotic changes that increase the risk of atrial fibrillation (AF) and ventricular tachycardia.

### 4.1 Intermittent Hypoxia

**Fig. 4.**
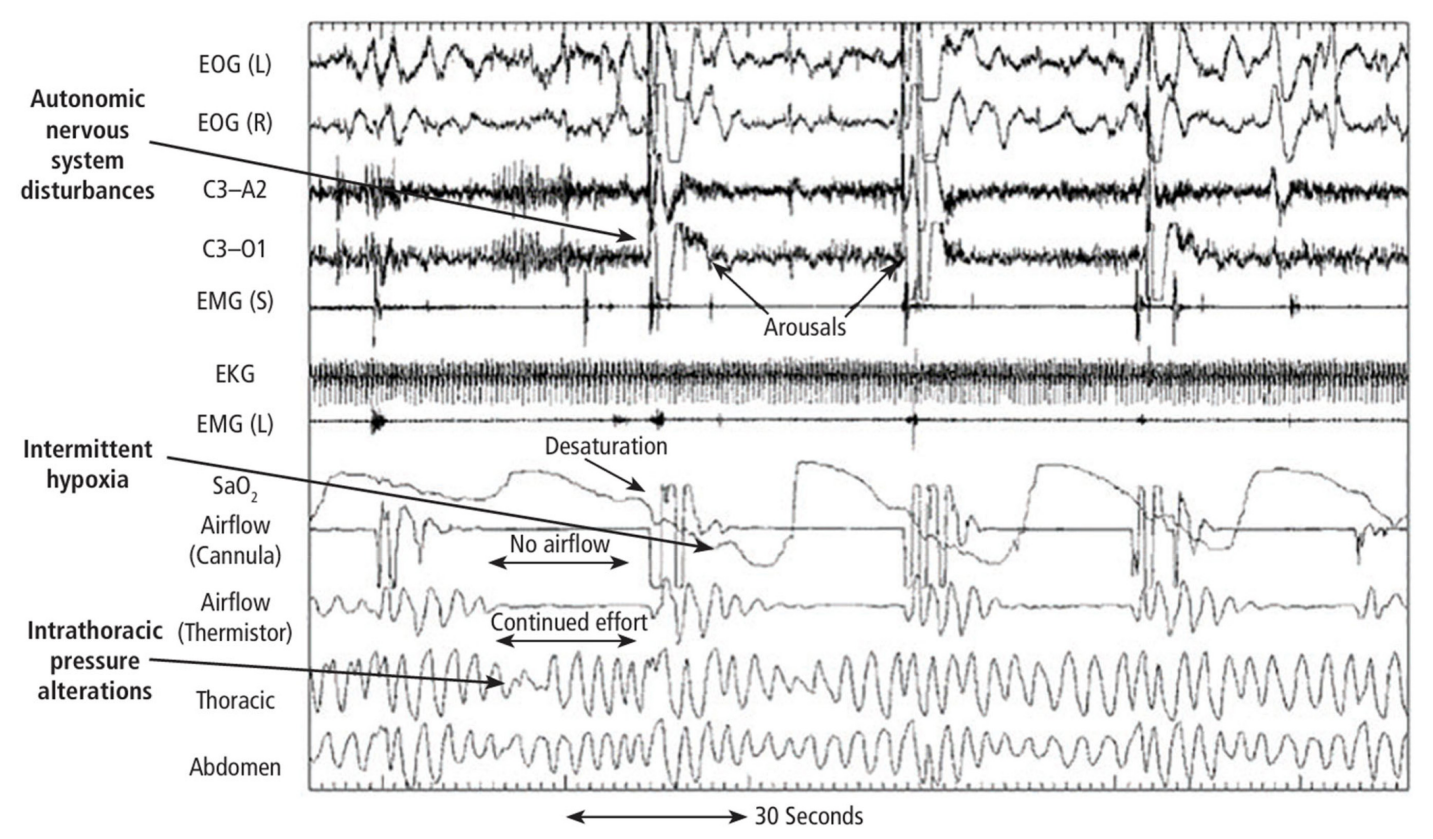
**A polysomnogram of the relationship between autonomic nervous system disturbances, intermittent hypoxia, and fluctuations in intrathoracic pressure that influence sleep-disordered breathing (SDB)**. This interplay negatively impacts airway patency through increased pharyngeal collapsibility and oronasal airflow turbulence, leading to a hypoxic state. The subsequent negative effects on respiratory effort and oxygen saturation are key features of OSA. EOG, electrooculogram for left (L) and right (R); C3-A2 and C3-O1, brain waves recorded via electroencephalogram; EMG, electromyogram for sleep (S) and leg (L) movement; EKG, electrocardiogram; SaO_2_, oxygen saturation. This image is from reference [16]*.*

Sleep fragmentation and increased respiratory effort contribute to the creation of an intermittent hypoxic (IH) state (Fig. 4, Ref. [16]), characterized by repeated oxygen desaturation–resaturation cycles, and is one of the most important causative factors for CVD complications associated with metabolic-related OSA [14]. Indeed, OSA causes recurrent collapse of the upper oropharyngeal airway during sleep, leading to repeated hypoxic episodes and intermittent oxygen desaturation and resaturation, thereby altering cellular physiology and adversely affecting biochemical and molecular pathways. Upper airway collapse is common in obese patients and in those with high fat deposition adjacent to the pharynx, which narrows and obstructs the airway. This obstruction causes a significant drop in intrathoracic pressure to approximately –60 mm Hg, leading to a drastic increase in transmural pressures across all cardiac chambers and great vessels [17].

The decrease in intrathoracic pressure increases venous return and preload, which causes right ventricular (RV) distension and a leftward shift of the interventricular septum, leading to decreased left ventricular (LV) filling in the setting of increased afterload, causing an overall decrease in stroke volume (SV) [15,18]. The change in this hemodynamic state, combined with hypoxia, causes sympathetic overactivation and stimulates the sympathetic nervous system (SNS), thereby increasing myocardial oxygen demand and the risk of ischemia and arrhythmia [15].

Animal studies investigating intermittent hypoxia (IH) in OSA have shown that IH induces cellular and tissue injury by increasing reactive oxygen species (ROS) formation and promoting inflammatory pathways. Meanwhile, subsequent work has demonstrated that these processes contribute to vascular dysfunction, transcriptional reprogramming, inflammation, and innate and adaptive immune activation, all of which can lead to cardiovascular morbidity and mortality [19]. Oxidative stress promotes sympathetic overactivation, cellular and systemic inflammatory cascades, and vascular comorbidities in patients with OSA [19].

ROS, generated during IH episodes, drive oxidative stress by inducing mitochondrial dysfunction, activating nicotinamide adenine dinucleotide phosphate (NADPH) oxidase (NOX) and xanthine oxidase (XOX), and causing nitric oxide synthase (NOS) uncoupling. Thus, NOS produces superoxide anions instead of its normal physiological product, nitric oxide (NO) [19]. The lack or underproduction of NO due to impaired NOS function promotes hypertension, inflammation, endothelial dysfunction, hypercoagulability, and atherosclerosis [19].

### 4.2 Sympathetic Overactivation

Sympathetic overactivation plays a crucial role in increasing ROS-dependent chemical responses, such as in angiotensin II and endothelin-1 levels, which also contribute to hypertension; meanwhile, ROS upregulate numerous redox-sensitive transcription factors such as nuclear factor-κB (NF-κB), hypoxia-inducible factor-1α (HIF-1α), and nuclear factor erythroid-derived 2-like 2 (NF2L2) [19]. NF-κB is involved in numerous biochemical processes, including the inflammatory response, ultimately leading to endothelial dysfunction and atherosclerosis, whereas HIF-1α and NF2L2 are vital protective mechanisms that are upregulated in response to increased ROS levels and act to counterbalance ROS-mediated injury [19].

Repetitive cycles of hypoxemia and hypercapnia cause excessive activation of both cardiac parasympathetic pathways and the SNS. During apnea termination, asphyxia triggers cortical arousal, during which the individual briefly awakens. This is accompanied by a surge in catecholamines, resulting in acute increases in blood pressure (BP) and heart rate (HR) [20]. Sympathetic overactivation in OSA also involves renal pathophysiology that negatively affects the cardiovascular system. OSA overstimulates the RAAS, leading to increased sodium reabsorption in the kidneys and vasoconstriction of the peripheral vasculature, thereby increasing vascular resistance and afterload [21]. Cardiac output (CO) remains elevated and becomes hyperdynamic in the setting of a decreased SV. The HR increases in response to decreased SV to maintain adequate CO and tissue oxygenation and perfusion.

Chronic states of sympathetic overactivation can lead to hypertension, endothelial dysfunction, arterial stiffness, and increased rigidity of peripheral vessels [21]. Myocardial stress, cardiac remodeling, and LV hypertrophy (LVH) are additional adverse consequences of sustained sympathetic drive and can contribute to chronic kidney disease, atherosclerosis, HF, and premature mortality [21]. In the myocardium, increased preload due to decreased intrathoracic pressure and the elevated afterload resulting from systemic hypertension ultimately leads to cardiomyocyte apoptosis, impaired tissue perfusion, myocardial fibrosis, and disruption of the cardiomyocyte cytoskeleton [21]. This maladaptive process is reinforced by a positive feedback loop characterized by increased intracellular calcium consumption in cardiomyocytes, heightened cardiac contractility, and increased myocardial oxygen demand [21].

### 4.3 Endothelial Dysfunction and Vascular Inflammation

The endothelium consists of a thin layer of endothelial cells that lines the inner surface of all blood and lymphatic vessels and is in direct contact with circulating blood, lymph, cells, proteins, and other intravascular molecules [22]. The functions of the endothelium include regulating blood fluidity, platelet aggregation, vascular tone, immune responses, inflammatory processes, angiogenesis, and, to some extent, hormone secretion and regulation [22]. Numerous variables, including genetics, obesity, sex, smoking, and other harmful agents and toxic cellular byproducts, such as oxidative stress, metabolic alterations, inflammation, and pollution, can lead to endothelial dysfunction by decreasing vasodilatory capacity, increasing proinflammatory and prothrombotic responses, and triggering abnormal vascular growth [23].

During early endothelial injury, proinflammatory molecules such as interleukin-1 (IL-1), interleukin-6 (IL-6), tumor necrosis factor alpha (TNF-α), and C-reactive protein (CRP) are released, causing endothelial dysfunction and increasing the expression of cell adhesion molecules (CAMs) such as E-selectin, vascular cell adhesion molecule-1 (VCAM-1), and intercellular adhesion molecule-1 (ICAM-1) [24]. IH has also been shown to impair endothelial function in animal models by depleting the endothelial progenitor cell (EPC) population in the blood [25]. EPCs are circulating bone marrow-derived precursors that function as excreting microvesicles (MVs) containing gene messages (mRNAs and miRNAs), which can exert either beneficial or detrimental effects on endothelial cells, depending on the presence or absence of specific environmental molecular markers [25]. For example, MVs in a medium containing TNF-α can activate caspase-3, thereby inducing apoptosis, whereas in other circumstances, MVs can stimulate cellular signaling cascades that increase ROS production, induce angiogenesis, and activate PI3K/eNOS/NO pathways [26].

In one study, MVs from patients with chronically stable oxygen desaturation were injected into animal models. These MVs impaired endothelium-dependent relaxation of vascular smooth muscle cells (VSMCs) in the aorta and reduced flow-mediated dilation (FMD) in small mesenteric arteries, mainly due to decreased NO production [25]. MVs from the same patient population also enhanced phosphorylation of endothelial nitric oxide synthase (eNOS), which was associated with increased caveolin-1 expression, a membrane protein that regulates eNOS activity and participates in insulin signaling, thereby decreasing NO bioavailability [25]. Interestingly, studies have shown that after CPAP treatment, NO levels and the sirtuin protein SIRT1 increase, with SIRT1 playing a significant role in the protective effects against oxidative stressors, ischemia–reperfusion injury, pathological cardiomyocyte remodeling, and myocardial infarction (MI) [25]. Normally, FMD increases in response to higher blood flow, reflecting physiological vasodilation.

### 4.4 Baroreceptor Dysfunction

OSA has been shown to modulate parasympathetic tone by altering baroreceptor function [27]. The autonomic effects of OSA are exacerbated by obesity and hypertension [28,29]. As described earlier, patients with these conditions often exhibit a higher sympathetic tone than individuals without OSA. Interestingly, in response to phenylephrine administration, sympathetic nerve activity was similar between the two groups [27]. In another study, non-pharmacological activities that increase BP, such as Valsalva and handgrip, produced exaggerated decreases in baroreceptor sensitivity among participants with OSA [30]. Conversely, following administration of an agent that suppresses baroreceptor activity, patients with OSA demonstrated less blunting of sympathetic nerve activity than healthy subjects [27]. Overall, baroreceptor modulation in OSA results in elevated sympathetic activity. Consequently, BP rises, and the capacity to normalize BP is impaired due to autonomic imbalance.

### 4.5 Chronotropic Incompetence

A diminished chronotropic response to exercise is one of the main mechanisms underlying exercise intolerance in OSA. Downregulation of β1-adrenergic receptors from chronic sympathetic stimulation has been proposed as a key driver of this phenomenon [31]. Indeed, a meta-analysis demonstrated that individuals with OSA have lower peak oxygen consumption and peak HR during exercise compared with controls [32]. Moreover, OSA severity correlates with impaired normalization of HR following exercise [33,34]. This inability to maintain a regular HR is also present during sleep [35]. While less pronounced than during exercise, evidence of inappropriate HR variability during sleep strongly suggests evolving autonomic dysfunction in OSA [35].

## 5. Cardiovascular Complications

### 5.1 Arrhythmias

#### 5.1.1 Atrial Fibrillation

The relationship between OSA and AF is complex and mediated by metabolic, inflammatory, autonomic, and neurohumoral mechanisms [36]. Observational studies have concluded with high certainty that OSA is a modifiable risk factor for recurrent AF and that treatment with CPAP reduces the incidence of AF recurrence after cardioversion or catheter ablation [37]. Up to 85% of individuals with nonvalvular AF have an underdiagnosed OSA comorbidity [38]. Meanwhile, OSA is likely to remain undetected in patients with AF, especially in nonobese and/or female patients [36]. A study from Toronto, Canada, by Abumuamar et al. [36] further highlighted the complexity of the relationship between OSA and AF in 100 patients with AF: 27% of patients with a normal overall AHI exhibited increased AHI during REM sleep, highlighting the elevated risk associated with undiagnosed OSA in patients with established AF.

**Fig. 5.**
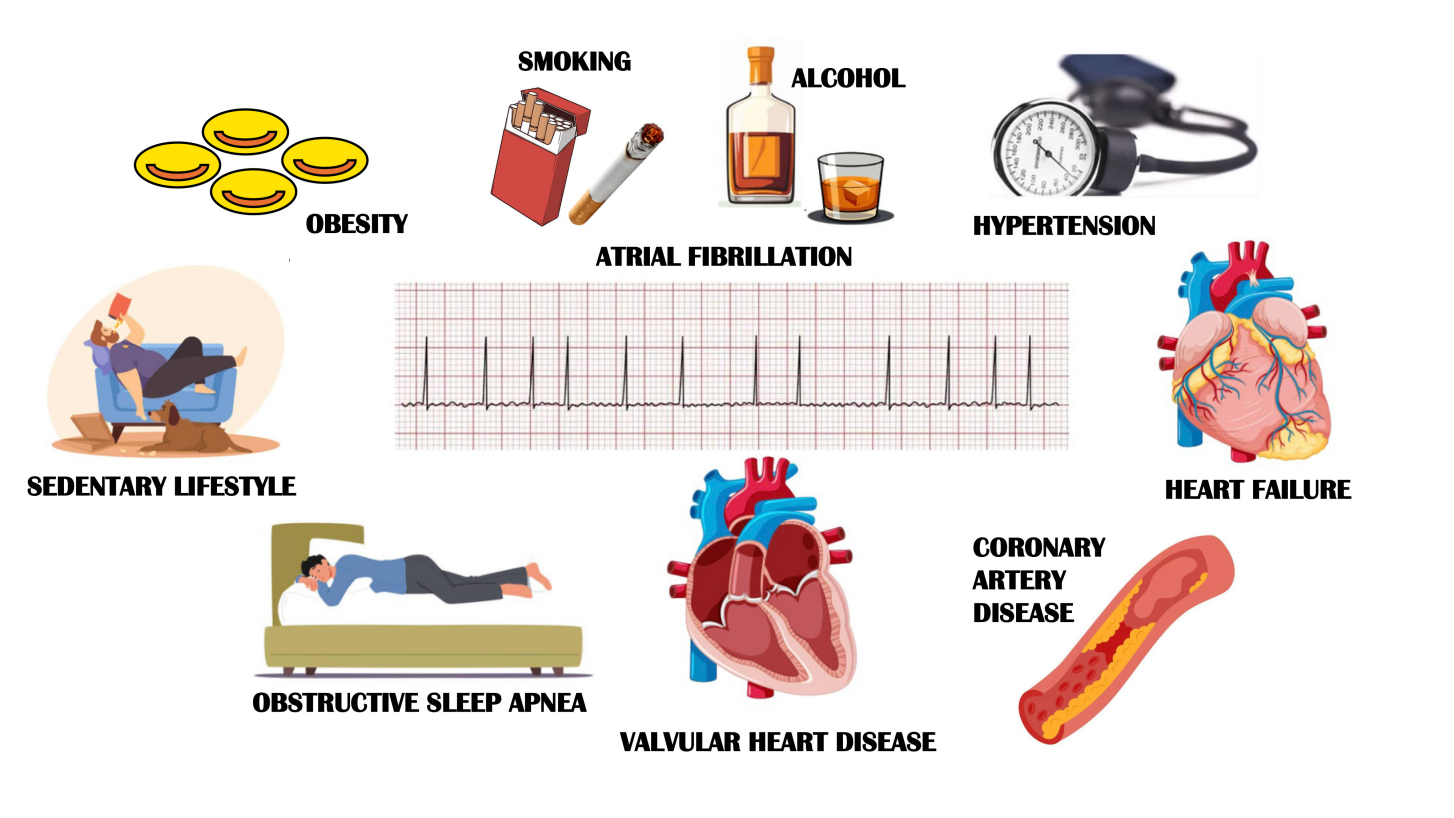
**Multifactorial factors that contribute to the development of atrial fibrillation**.

Data also suggest that patients with OSA have a higher incidence of non-pulmonary-vein triggers for AF, and that ablation of these additional triggers reduces the risk of AF recurrence compared with patients whose AF is driven solely by pulmonary vein triggers [37]. Structural and electrical atrial changes have also been studied in OSA patients with AF. Structural changes include increased atrial size, concomitant extensive low-voltage areas, and regions of electrical silence due to loss of atrial myocardium, likely secondary to fibrosis [39]. These alterations reflect pathologic atrial remodeling that disrupts normal atrial architecture and leads to electrophysiologic abnormalities, including reduced voltage, focal and diffuse conduction disturbances, and prolonged sinus node recovery time, all of which promote the initiation and progression of AF [39]. Although the cause of AF is multifactorial and can include alcohol use, smoking, hypertension, and HF (Fig. 5), sleep apnea plays a crucial role in the creation of an arrhythmogenic state that can lead to AF.

#### 5.1.2 Ventricular Arrhythmias

Apart from AF, other arrhythmias can also occur in patients with OSA. Ventricular arrhythmias, such as premature ventricular contractions (PVCs), in bigeminy or trigeminy patterns, are more common in OSA than in patients without OSA (25% vs. 14.5%) [40,41]. A high PVC burden can lead to cardiac repolarization anomalies, increasing the risk of life-threatening ventricular arrhythmias, such as ventricular fibrillation [42]. Notably, the risk of sudden cardiac death is highest during sleep [43,44]. However, unlike other cardiac complications, OSA severity does not correlate with the frequency or severity of ventricular arrhythmias [45].

The mechanisms underlying ventricular tachycardia (VT) and sudden cardiac death (SCD) in sleep apnea are multifactorial. Acute triggers that create arrhythmogenic states arise from alterations in intrathoracic pressure during respiration, leading to left atrial stretch, distension, and changes in LV afterload, all of which can increase the incidence of atrial premature beats and prolong the QT interval [46]. Intermittent hypoxemia and hypercapnia further trigger excessive sympathetic activation, increasing the automaticity of ectopic foci [46]. Chronic hypertension contributes to causing left ventricular hypertrophy and cardiac remodeling, which promote fibrosis and an arrhythmogenic substrate.

#### 5.1.3 Bradyarrhythmias

Bradyarrhythmias, such as sinus bradycardia, sinus pauses, and Wenckebach block, are also observed in patients with OSA [41]. Sinus bradycardia has been reported in 7.2%–40% of patients with severe OSA; second- and third-degree AV block in 1.3–13.3%; sinus pauses in 3.3–33% [47]. The management of these arrhythmias focuses on treating the underlying OSA and involves a multidisciplinary approach to address risk factors and provide patient education [48].

#### 5.1.4 Torsades de Pointes

Patients with OSA also have a higher probability of developing prolonged QTc intervals on resting daytime electrocardiograms (EKGs), which predisposes these patients to life-threatening arrhythmias such as Torsades de Pointes (TdP) [49]. Notably, TdP is a polymorphic ventricular tachycardia that can cause syncope, seizures, CVAs, sudden cardiac arrest, and death without prompt medical intervention [49].

### 5.2 Heart Failure

CSA is caused by abnormal chemoreceptor sensitivity, resulting in a disruption of the coupling between the brainstem respiratory drive center and the respiratory muscles and leading to a temporary loss of ventilatory drive during sleep [50]. CSA is more strongly associated with complications related to HF, due in part to oscillatory ventilation patterns, with central apnea and hypopnea episodes caused by alternating hyperventilation phases termed Cheyne–Stokes respiration [50].

In OSA, inspiratory efforts against an occluded airway during apnea reduce intrathoracic pressure, whereas in CSA with HF, pulmonary congestion decreases lung compliance and increases the negative intrathoracic pressure generated during hyperpnea phases. These changes lead to several hemodynamic consequences, including increased preload, RV distention, and a leftward shift of the interventricular septum, which reduces LV filling and stroke volume [50].

### 5.3 Pulmonary Hypertension

Pulmonary hypertension (PH) is strongly associated with OSA in a bidirectional manner, with a reported prevalence as high as 70–80% among patients with PH confirmed by right-sided heart catheterization (RHC) [6]. The main pathophysiological mechanisms linking PH to OSA are driven by hypoxia-induced pulmonary arteriolar vasoconstriction, mediated by multiple molecular signaling and cellular pathways, including NO, endothelin, angiopoietin-1, serotonin, and NADPH oxidase [6].

Patients with OSA-related PH have increased hypoxia-induced pulmonary vasoconstriction due to chronic sustained hypoxia, which promotes vascular remodeling and irreversible cardiopulmonary hemodynamic changes, including increased pulmonary vascular resistance and RV dysfunction [51]. CPAP therapy has been shown to reduce hypoxic vascular reactivity, with observational studies demonstrating reductions in pulmonary artery pressure of approximately 5 mm Hg and in declines in pulmonary vascular resistance among patients with a confirmed diagnosis of PH who are adherent to CPAP [51].

### 5.4 Endothelial Dysfunction and Atherosclerosis

The common mechanism of endothelial dysfunction in OSA is intermittent hypoxia, which promotes the formation of ROS [52,53]. Notably, ROS suppresses NO bioavailability and promote lipid peroxidation, thereby increasing the risk of atherosclerosis [54]. Upregulation of cyclooxygenase-2 (COX-2) creates a proinflammatory state and induces vasoconstriction [52]. Additionally, proinflammatory markers such as interleukin-4 (IL-4) and IL-6 are elevated in individuals with moderate-to-severe OSA [55,56]. Inflammation in endothelial cells and vascular tissues causes significant damage, leading to atherosclerosis [56]. Abnormal endothelial function predisposes to cardiovascular complications such as hypertension, stroke, and CAD [52,53].

### 5.5 Coronary Artery Disease

The development of CAD in patients with OSA is multifactorial, involving endothelial dysfunction, oxidative stress, and inflammation [8,57,58]. Intermittent hypoxia is the principal driver of these pathologic processes [57,58]. Hypoxia-induced oxidative stress promotes endothelial injury and lipid peroxidation, ultimately leading to atherosclerotic plaque formation [59]. Furthermore, activation of inflammatory cytokines and C-reactive protein (CRP) contributes to the development of atherosclerosis. Among patients with acute coronary syndrome (ACS), OSA was found in 30% of patients in one study and 69% in another, underscoring how underdiagnosed OSA remains [60,61,62,63]. CAD severity, as indicated by elevated SYNTAX scores, correlates with OSA severity [53]. Nocturnal angina was also commonly associated with OSA among patients in a small observational study [64]. Additionally, in patients with ST-elevation myocardial infarction (STEMI), OSA was associated with higher rates of major adverse cardiac events (MACEs), including recurrent MI leading to revascularization and CVA [62,63]. Other adverse events, such as hospitalization and mortality, were also higher in patients with OSA [62,63].

### 5.6 Stroke

OSA is a common and important risk factor for strokes or CVAs. The risk of stroke increases with elevated AHI values [65,66]. Typically, an AHI >30 is associated with the highest risk of developing a CVA, with a hazard ratio of 2.52 among individuals aged 70–100 years [66]. This increased risk of stroke is independent of other risk factors such as hypertension, diabetes, and AF [67]. Moreover, an AHI >30 is associated with longer hospitalization and longer recovery times [68]. OSA also increases the risk of other common CVA risk factors, such as hypertension and diabetes, further exacerbating overall risk.

### 5.7 Type 2 Diabetes Mellitus

Type 2 diabetes mellitus (T2DM) is another non-cardiovascular complication of OSA. Recurrent hypoxemia combined with poor sleep quality can lead to insulin resistance and glucose metabolism dysfunction [69]. For example, one study found that 5 hours of intermittent hypoxia during sleep was associated with a 17% decrease in insulin sensitivity without a significant increase in compensatory insulin production [70]. Moreover, functional effects of OSA, such as daytime fatigue, further limit physical activity and exercise, increasing the risk of developing T2DM or worsening pre-existing T2DM. The mainstays of treatment for T2DM, such as weight loss and lifestyle changes, are also effective for OSA. Meanwhile, CPAP therapy has been shown to improve glucose metabolism, reduce blood pressure, and enhance insulin sensitivity [71].

### 5.8 Interactions of Comorbidities Independent of OSA

Obesity, diabetes, hypertension, and lifestyle factors such as smoking can have independent adverse effects on cardiovascular health that are not solely mediated by sleep apnea. Diabetes mellitus negatively affects cardiovascular health by damaging the microvasculature, leading to cardiovascular, cerebrovascular, and peripheral arterial complications and increasing rates of CAD, CVAs, and peripheral vascular disease [72]. Obesity is another public health epidemic that increases the risk of CVD through complex metabolic, endocrinological, immunological, structural, and hemodynamic mechanisms, leading to poor cardiovascular outcomes and increased morbidity and mortality. Genetic investigations into obesity have shown that each 1 kg/m^2^ increase in body mass index (BMI) is associated with higher risks of poor CVD outcomes (Table 2) [73].

**Table 2.** **Genetically linked association between an increase in body mass index of 1 kg/m^2^ and increased risk of cardiovascular-related complications**.

The relationship between an increasing BMI of 1 kg/m^2 ^& its associated cardiac & vascular complications
Aortic Valve Stenosis
Heart Failure
Deep Vein Thrombosis
Arterial Hypertension
Peripheral Artery Disease
Coronary Artery Disease
Atrial Fibrillation
Pulmonary Embolism
Subarachnoid Hemorrhage
Abdominal Aortic Aneurysm
Intracerebral Hemorrhage
Ischemic Stroke
Transient Ischemic Attack
Thoracic Aortic Aneurysm

## 6. Treatment and Management Strategies

**Fig. 6.**
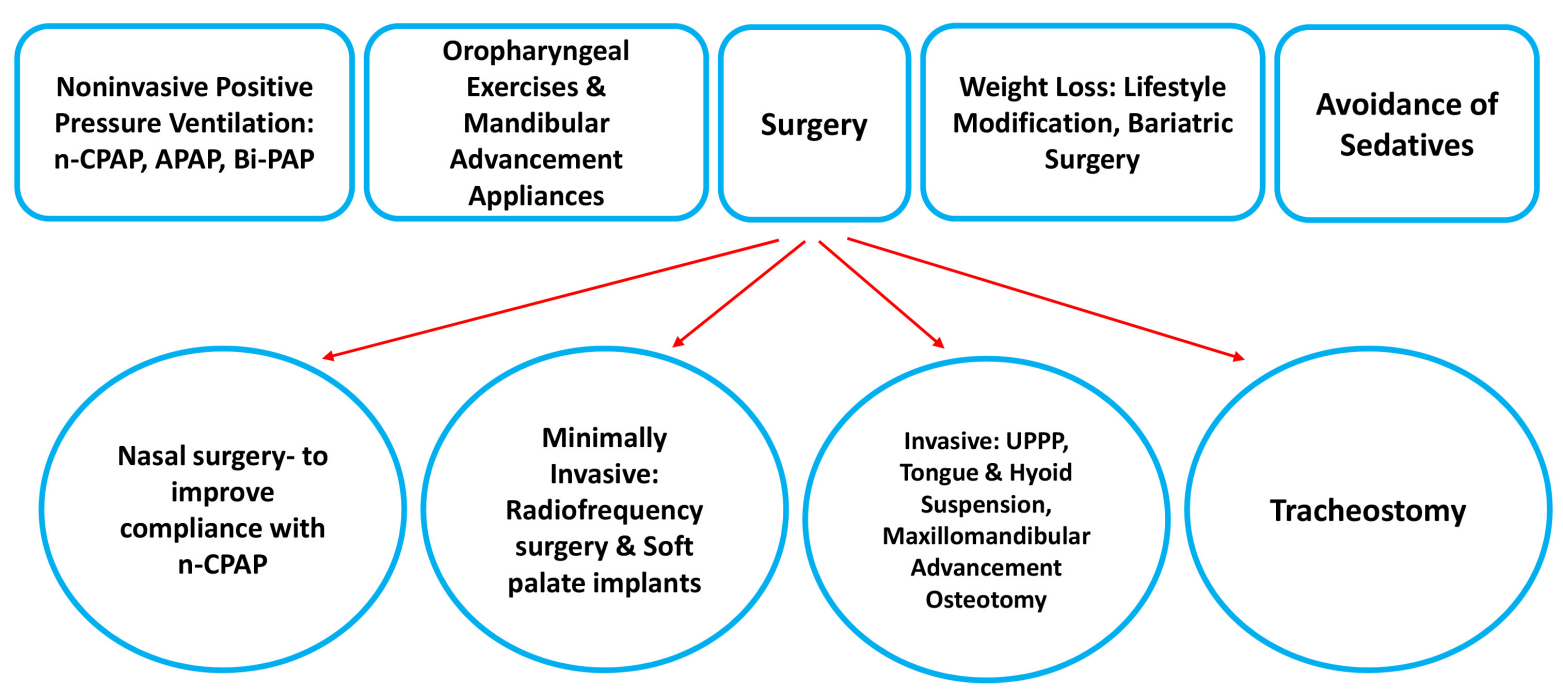
**Treatment modalities utilized in OSA**. n-CPAP, nasal continuous positive airway pressure; APAP, auto-titrating continuous positive airway pressure; Bi-PAP, bilevel positive airway pressure; UPPP, uvulopalatopharyngoplasty.

Several treatment options are available for OSA (Fig. 6), including CPAP, auto-titrating positive airway pressure (PAP), bilevel PAP (BiPAP), adaptive servo-ventilation (ASV), weight loss, dietary modifications, and lifestyle changes such as exercise. Additional modalities include positional therapy, oral appliances, upper airway surgery, upper airway neurostimulation, and bariatric surgery [6]. Further management strategies focus on treating CVD complications, including BP control. CPAP has been shown in numerous randomized controlled trials (RCTs) to be effective in reducing BP, with reductions of 2.5 mm Hg in 24-hour systolic and diastolic BP [71]. CPAP is especially effective in patients with resistant hypertension attributable to OSA, with reported decreases in systolic BP of 4.7–7.2 mm Hg and diastolic BP of 2.9–4.9 mm Hg [71]. Regarding HF, ASV has shown promise in decreasing morbidity and mortality related to HF. RCTs in patients with HFrEF have shown that CPAP treatment has significant cardiac effects, including reducing hypersensitivity of myocardial drive, reducing awake sympathetic activity, and increasing left ventricular ejection fraction (LVEF) by approximately 5% within 1 month of treatment initiation [71].

### 6.1 Therapeutic Modalities for Sleep Apnea

As mentioned previously, HF plays a crucial role in the CVD burden in association with uncontrolled SDB. Positive airway pressure (PAP) therapies remain the mainstay of OSA treatment, but the management of CSA in the context of HF continues to evolve. The presence of CSA in patients with HF is associated with worse prognosis and outcomes; thus, routine screening for signs and symptoms of CSA in patients with HF is vital and of utmost importance [74].

Organized exercise training programs and increased physical activity in patients with CSA and HF have been shown to decrease symptomatic burden and reduce complications related to the sequelae of CVD. In one study, 18 patients with chronic HF who participated in a six-month course of structured aerobic exercise training significantly decreased the number of CSAs per night [75]. Initially, the Canadian Continuous Positive Airway Pressure for Patients with CSA and HF (CANPAP) trial showed that LVEF improved and transplant-free survival rates were positively impacted in 57% of patients in whom the AHI was reduced to <15; however, the study was limited by insufficient statistical power and poor patient compliance to device use [74].

The ASV device, designed to deliver adequate positive pressure to maintain ventilation, has shown mixed results in extending benefits for HF patients with CSA or those unresponsive to CPAP. Earlier studies showed that ASV decreased AHI and improved LVEF; however, the subsequent large multicenter Treatment of Predominant Central Sleep Apnea by Adaptive Servo-Ventilation in Patients with Heart Failure (SERVE-HF) trial showed a paradoxical increased risk of cardiovascular mortality with ASV in CSA-HFrEF patients, potentially due to compromised cardiac output from PAP [74].

Results from the Cardiovascular Improvements with MV-ASV Therapy in Heart Failure (CAT-HF) trial showed no improvement in 6-month outcomes among patients receiving ASV in addition to optimized medical therapy (OMT), compared with OMT alone [76]. The ASV for SDB in Patients with HFrEF (ADVENT-HF) trial found that ASV significantly reduced the AHI in patients with HF and sleep apnea; however, the trial did not meet the primary endpoint of reducing cardiovascular risk, including hospitalization, arrhythmias such as AF, and death [77].

Other non-PAP treatment modalities for CSA patients include cardiac resynchronization therapy, which can reduce AHI events in patients with CSA and HFrEF, and nocturnal oxygen therapy, which can attenuate the ventilatory response to carbon dioxide (CO_2_). Neurostimulation with the Remedē ^(^™^)^ system (ZOLL Respicardia, Minnesota), which is an implantable lead-based device positioned in the left or right pectoral region, has gathered considerable interest in mitigating the complications related to CSA. The implant enables unilateral transvenous phrenic nerve stimulation (TPNS) during sleep, causing diaphragmatic contraction that mimics the normal physiological respiratory drive seen in patients without CSA [74]. In a multicenter study evaluating the Remede^ (TM)^ system, 73 of 151 eligible patients (majority of which had a history of HF) showed improvement in central apnea index and quality of life [78]. At 6 months, a greater proportion of patients receiving the TPNS implant had an AHI reduction of ≥50% from baseline than in the control group [78].

### 6.2 Pharmacological Intersections and Considerations

OSA frequently intersects with cardiovascular pharmacotherapy, creating both challenges and opportunities for management. β-blockers mitigate the sympathetic surges and nocturnal BP variability that characterize OSA. Cardioselective β-blockers are safe, do not worsen apnea severity, and may improve nocturnal BP control in hypertensive OSA cohorts [79,80,81]. Although antihypertensive medications generally exert only modest effects on the AHI, β-blockers remain integral to cardiovascular protection in patients with OSA.

Diuretics and mineralocorticoid receptor antagonists (MRAs) are important in addressing fluid-mediated mechanisms of OSA. Rostral fluid shift during sleep contributes to upper airway narrowing. Intensified diuretic therapy and spironolactone have been shown to reduce AHI, morning BP, and neck circumference, particularly in patients with resistant hypertension and OSA [82,83,84,85]. These therapies complement CPAP by targeting distinct mechanisms of BP dysregulation and airway collapsibility. More recently, sodium–glucose cotransporter-2 inhibitors (SGLT2i) have been proposed as potential modulators of SDB. By promoting natriuresis, osmotic diuresis, and weight loss, SGLT2i may attenuate airway obstruction and improve oxygenation. Early observational and pilot studies in diabetes and HF cohorts suggest favorable effects on AHI and oxygen saturation, although robust randomized data are not yet available [86,87,88].

Several agents remain under investigation. Acetazolamide, a carbonic anhydrase inhibitor, induces a mild metabolic acidosis that stimulates ventilatory drive. Randomized studies have demonstrated significant reductions in AHI, arterial stiffness, and nocturnal BP in patients with OSA and hypertension [89,90]. Theophylline, a respiratory stimulant, has reduced the burden of central apnea in small HF–CSA trials but has a narrow therapeutic index and proarrhythmic risk, limiting its clinical utility [91,92].

Pharmacotherapy achieves its greatest impact when combined with device-based therapy. In resistant hypertension, CPAP provides clinically relevant reductions in 24-hour BP, especially in nondipper phenotypes, and works synergistically with diuretics and MRAs [93,94]. In secondary prevention, the Survival and Ventricular Enlargement (SAVE) trial showed neutral effects of CPAP on major cardiovascular events in patients with established CVD, largely attributable to poor adherence (mean 3.3 h/night). Importantly, CPAP improved symptoms and quality of life, and adherent subgroups experienced fewer recurrent events [95]. In HFrEF, CPAP improves left ventricular function, sympathetic tone, and afterload, thereby enhancing the benefits of guideline-directed medical therapy (GDMT), including β-blockers, MRAs, angiotensin receptor–neprilysin inhibitors (ARNIs), and SGLT2i [96]. In contrast, adaptive servo-ventilation is contraindicated in HF with predominant CSA due to increased mortality observed in the SERVE-HF trial [97]. Finally, CPAP use in AF also reduces recurrence after catheter ablation and supports rhythm-control strategies when combined with antiarrhythmic drugs [98,99]. Collectively, these findings underscore that pharmacological agents and CPAP should be conceptualized as synergistic rather than isolated strategies, with therapy tailored to patient phenotype and adherence.

Other trials investigating the impact of CPAP on sleep apnea and cardiovascular outcomes are less convincing. For example, the Swedish Randomized Intervention with CPAP in Coronary Artery Disease and Obstructive Sleep Apnea (RICCADSA) trial targeted a CAD population to determine whether CPAP reduces the risk of long-term adverse cardiovascular outcomes in patients with CAD and non-sleepy OSA. The RICCADSA trial showed no significant reduction in cardiovascular events with CPAP in patients with CAD and OSA [100]. Another multicenter study, completed in 2022, evaluated CPAP in older adults with moderate-to-severe OSA. This study showed that CPAP effectively reduced the AHI and snoring in patients aged over 80 years; however, CPAP often failed to significantly improve daily symptoms, quality of life, or BP values compared with younger cohorts [101]. This study is significant because the findings suggest that the potential benefits of CPAP may plateau and become attenuated beyond approximately 80 years of age. Similarly, in the SAVE trial, CPAP plus usual care, compared with usual care alone, did not reduce cardiovascular events in patients with moderate-to-severe OSA and pre-existing CVD [95].

Meanwhile, new pharmacological therapies, including some glucagon-like peptide-1 receptor agonists (GLP-1 RAs), such as tirzepatide, have recently been approved by the United States Food and Drug Administration (FDA) for the treatment of OSA [102]. Tirzepatide was approved following the SURMOUNT-OSA trial, which showed that this medication significantly reduces the severity of sleep apnea by markedly lowering the AHI and reducing body weight in obese individuals. Indeed, in patients with moderate-to-severe OSA and obesity, tirzepatide reduced the AHI, body weight, hypoxic respiratory burden, and systolic BP [103]. The SURMOUNT-OSA trial also demonstrated significant disease modification in patients receiving tirzepatide, along with improvements in secondary endpoints, including reductions in high-sensitivity C-reactive protein (hs-CRP), an indicator of inflammatory response, and further inhibition of intrinsic cellular and molecular damage [103].

## 7. Future Directions

As discussed earlier, there is potential benefit to using CPAP in mitigating or even treating the cardiovascular complications of OSA. RCTs have investigated the hypothesis that CPAP can prevent the development of CVD. In one trial, CPAP significantly reduced hypertension compared with control (mean systolic BP 143 mm Hg vs. 139 mm Hg; *p* = 0.043), but did not affect MACEs [104]. The Impact of Sleep Apnea Syndrome in the Evolution of Acute Coronary Syndrome (ISAACC) trial compared CPAP therapy for ACS with concomitant OSA versus standard care [105]. At the end of the 3-year follow-up, the rates of MACEs were similar between the two groups (16% vs. 17%) [105]. Additionally, strict CPAP adherence did not reduce the risk of MACEs [105]. However, as in the previous study, a modest improvement in BP was observed in the intervention group [105]. Since the evidence does not support primary or secondary prevention of MACEs with CPAP, more RCTs examining MACE prevention with other OSA treatments are warranted.

Recently, an RCT evaluated the efficacy of uvulopalatopharyngoplasty (UPPP) combined with tongue reduction. The trial found that surgical intervention significantly reduced the AHI from 47.9 at baseline to 20.8 at 6 months [106]. Additionally, more patients in the intervention group reported better sleep quality than those in the medical management group [106]. However, UPPP with tongue reduction did not affect BP, and the intervention group experienced more adverse effects than the control group [106]. While UPPP with tongue reduction is promising for patients who cannot tolerate CPAP therapy, the efficacy of this procedure in preventing MACEs has yet to be elucidated.

Mandibular advancement devices (MADs) are a less invasive and more easily accessible treatment for sleep apnea. A meta-analysis found that MADs can reduce systolic BP by a mean of 2 mm Hg compared with control [107]. Meanwhile, when compared to CPAP, there is no difference in systolic BP reduction between the two devices [107]. Currently, no RCTs or meta-analyses have examined the primary or secondary prevention of MACEs with MADs.

Neurostimulation is the latest therapeutic modality for OSA. Phrenic nerve stimulation (PNS) and hypoglossal nerve stimulation (HNS) are the two available approaches. An RCT supported the efficacy of PNS, with a significantly higher proportion of patients achieving a >50% reduction in AHI compared with the control group at 6-month follow-up [78]. Moreover, patients treated with PNS had fewer hypoxic events (O_2_ saturation <90%) during sleep [78]. However, this trial has limited external validity because most participants were Caucasian males [78]. Cardiovascular outcomes such as MACEs and BP reduction were not assessed [78].

For HNS, while the therapy effectively reduced AHI compared with the control group, the approach had little effect on cardiovascular outcomes [108]. Mean 24-hour systolic BP was similar between the HNS and control groups (122.8 mm Hg vs. 123 mm Hg, respectively) [108]. Other cardiovascular markers, such as pre-ejection period and flow-mediated dilation, were also comparable, indicating that HNS confers no significant benefit in reducing sympathetic tone (which would be expected to shorten the pre-ejection period) and no effect on NO production (which is inhibited in OSA and contributes to impaired arterial dilation) [108]. Overall, while nerve stimulation is an effective, minimally invasive therapy for treating OSA in patients who cannot tolerate or have failed CPAP, the benefit of this approach in preventing MACEs has yet to be determined.

## 8. Conclusion

OSA is increasingly recognized as a major and modifiable contributor to CVD. Strong evidence links sleep apnea to hypertension, CAD, HF, atrial and ventricular arrhythmias, pulmonary hypertension, stroke, and sudden cardiac death. The pathophysiological and mechanistic interplay between sleep apnea and CVD centers on the interaction among intermittent hypoxia, sympathetic overactivity, a proinflammatory state, and oxidative and hemodynamic stress [109]. Collectively, these processes exacerbate cardiovascular morbidity and mortality and also affect other organ systems [109]. Early recognition of sleep apnea is critical. Screening strategies such as the STOP-BANG questionnaire, which assesses key risk factors for OSA, including snoring, tiredness, observed apneas, BP, BMI, age, neck circumference, and sex, coupled with timely referral for sleep testing, facilitate diagnosis in at-risk populations. Since the cardiovascular impact of sleep apnea spans multiple disease states, an interdisciplinary treatment framework integrating cardiology, sleep medicine, primary care, and behavioral health is essential for optimizing outcomes.

CPAP remains the cornerstone therapy, with proven benefits in symptom control, BP regulation, and arrhythmia prevention. However, CPAP alone has yielded mixed results in large cardiovascular outcome trials, highlighting the importance of adherence and the need for individualized management. Integration of pharmacologic agents (*e*.*g*., diuretics, MRAs, SGLT2i, acetazolamide) tailored to pathophysiologic phenotype, alongside device-based therapy, represents an evolving strategy that may enhance outcomes in select subgroups. Despite decades of research, critical knowledge gaps remain. Long-term RCTs are needed to clarify the impact of sleep apnea treatment on hard cardiovascular endpoints, to define optimal combinations of pharmacological and device-based therapies, and to identify which patient phenotypes derive the greatest benefit. Addressing these gaps will be essential to moving from symptom relief toward true cardiovascular risk reduction in patients with sleep apnea.
